# Supplementary figures and images for: Towards minimizing efforts for Morphing Attacks—Deep embeddings for morphing pair selection and improved Morphing Attack Detection
Source: PLoS One. 2024 May 31;19(5):e0304610. doi: 10.1371/journal.pone.0304610 (PMC11142600; doi:10.1371/journal.pone.0304610)

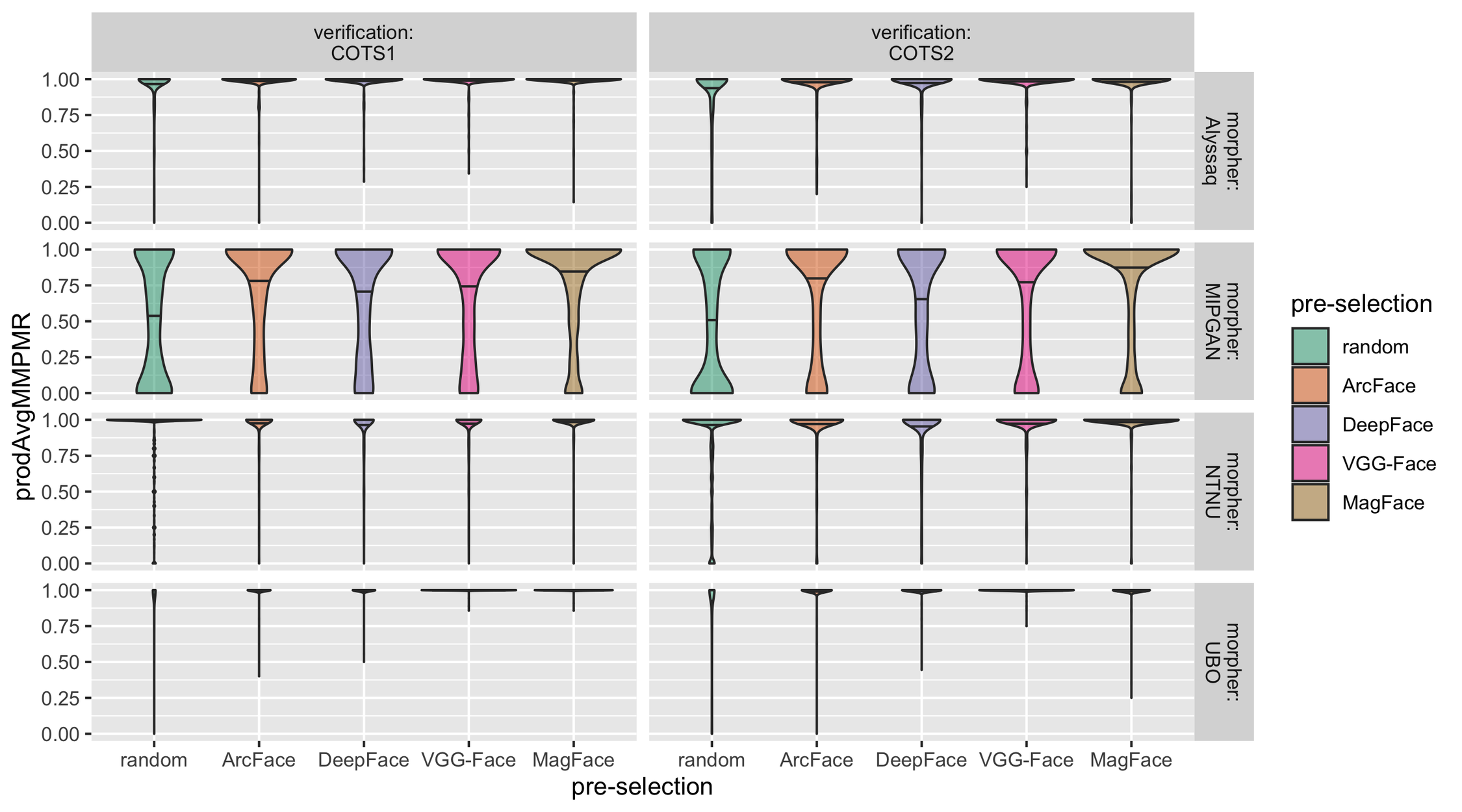

Supplement: S1 Fig — All morphs have been evaluated by different COTS FRSs (columns). See Fig 5 for details. (TIF) [file pone.0304610.s001.tif]

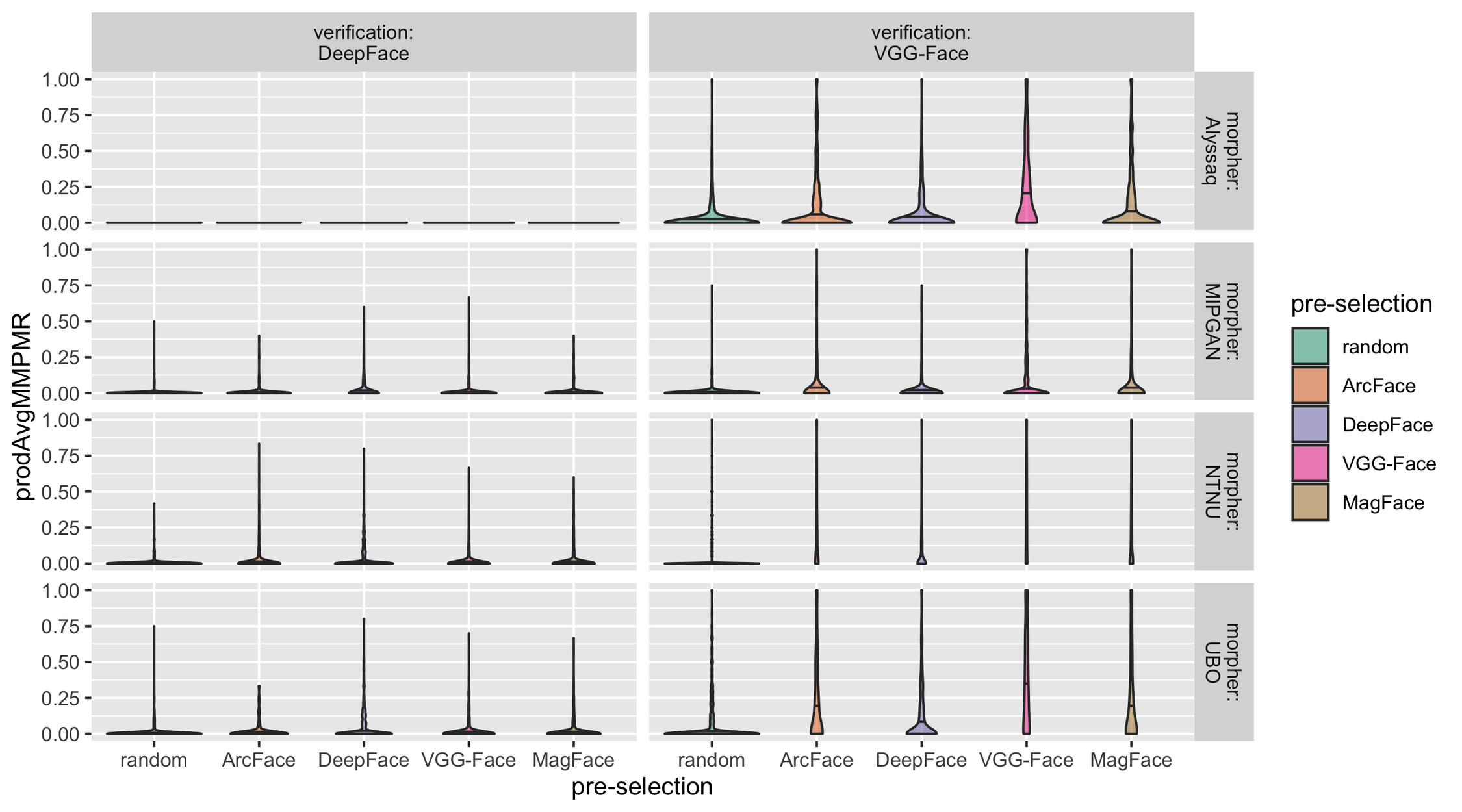

Supplement: S2 Fig — All morphs have been verified using DeepFace and VGG-Face (columns). See Fig 5 for verifications using ArcFace and MagFace and for more details. (TIF) [file pone.0304610.s002.tif]

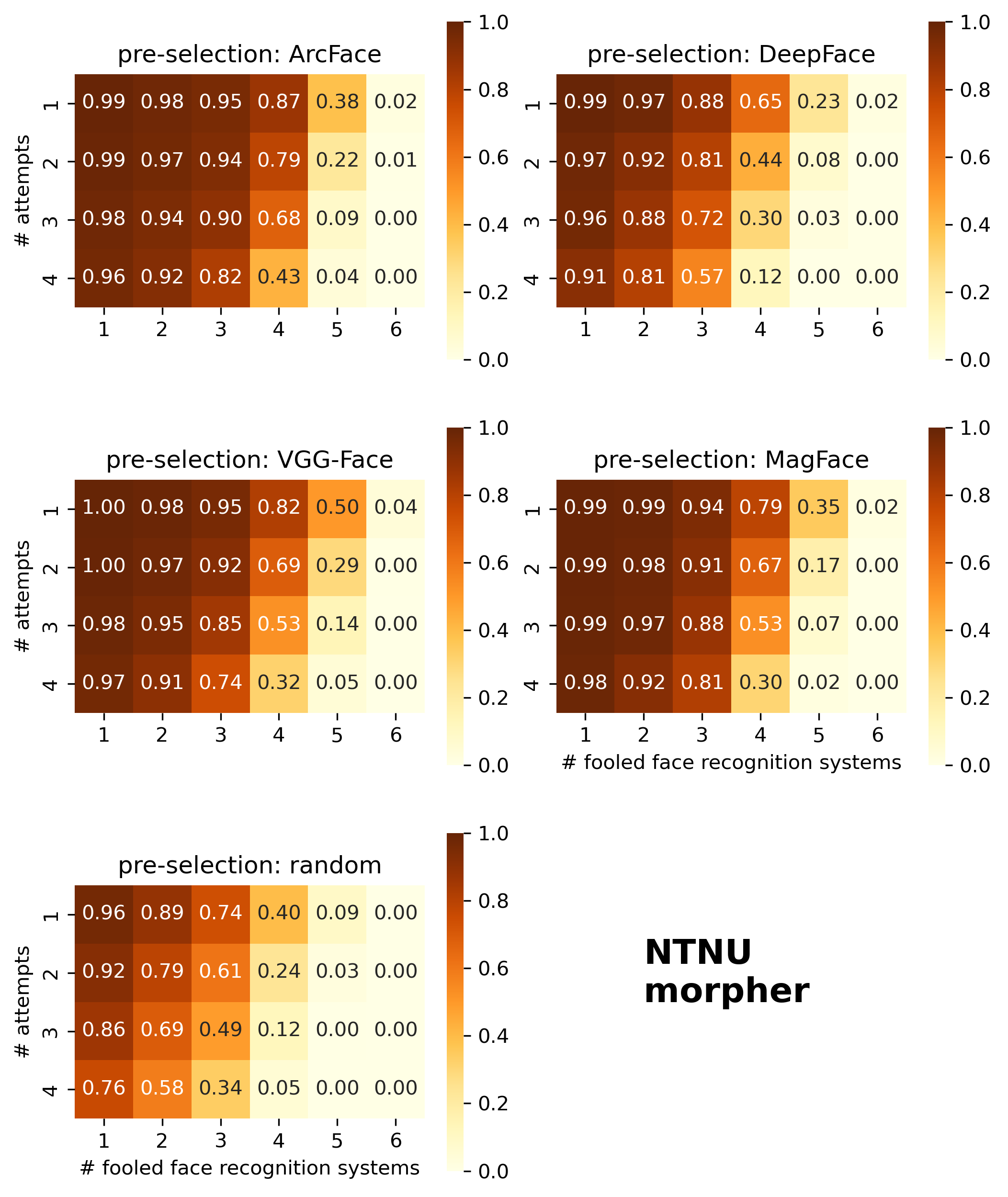

Supplement: S3 Fig — See Fig 6 for details. (TIF) [file pone.0304610.s003.tif]

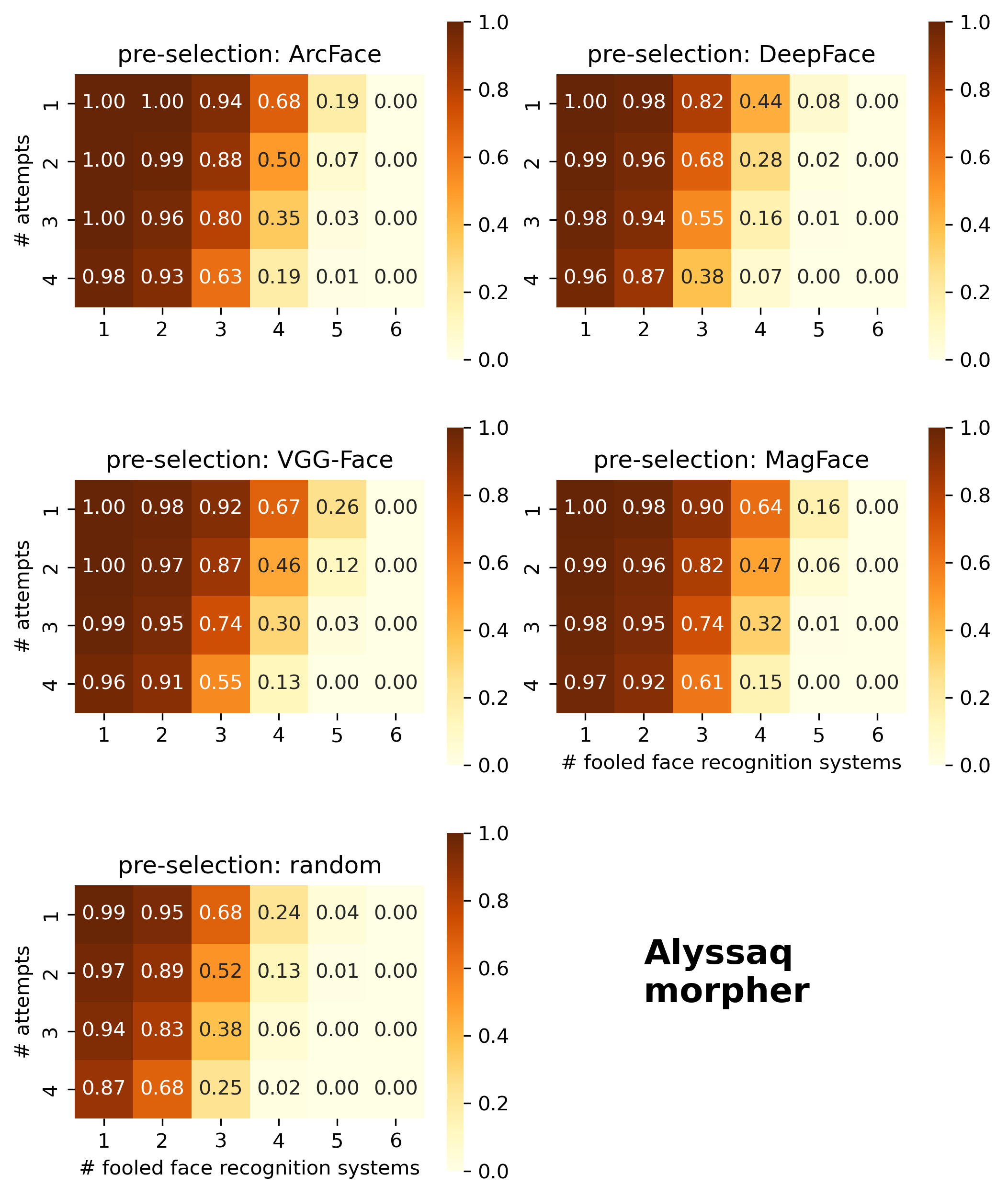

Supplement: S4 Fig — See Fig 6 for details. (TIF) [file pone.0304610.s004.tif]

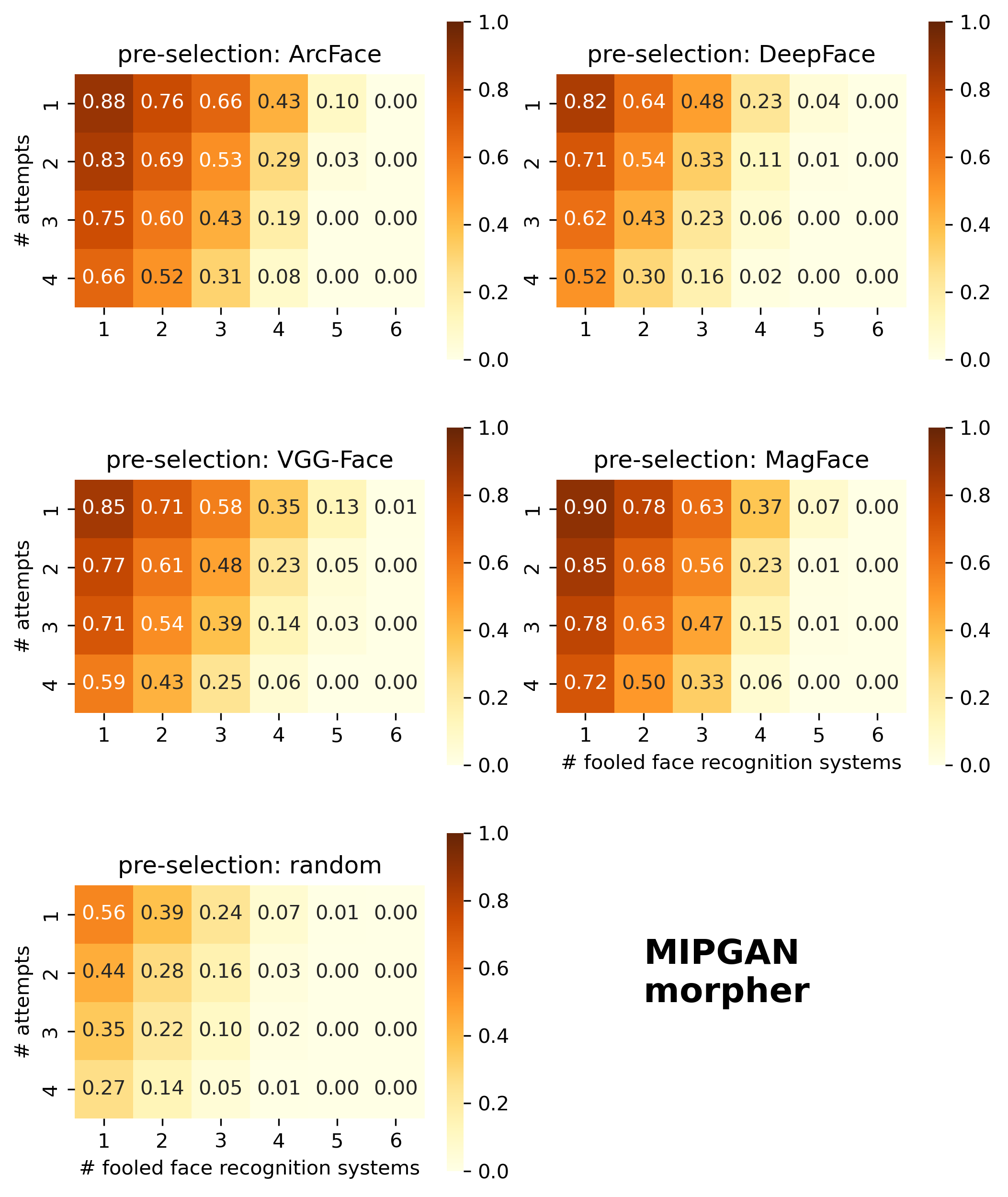

Supplement: S5 Fig — See Fig 6 for details. (TIF) [file pone.0304610.s005.tif]

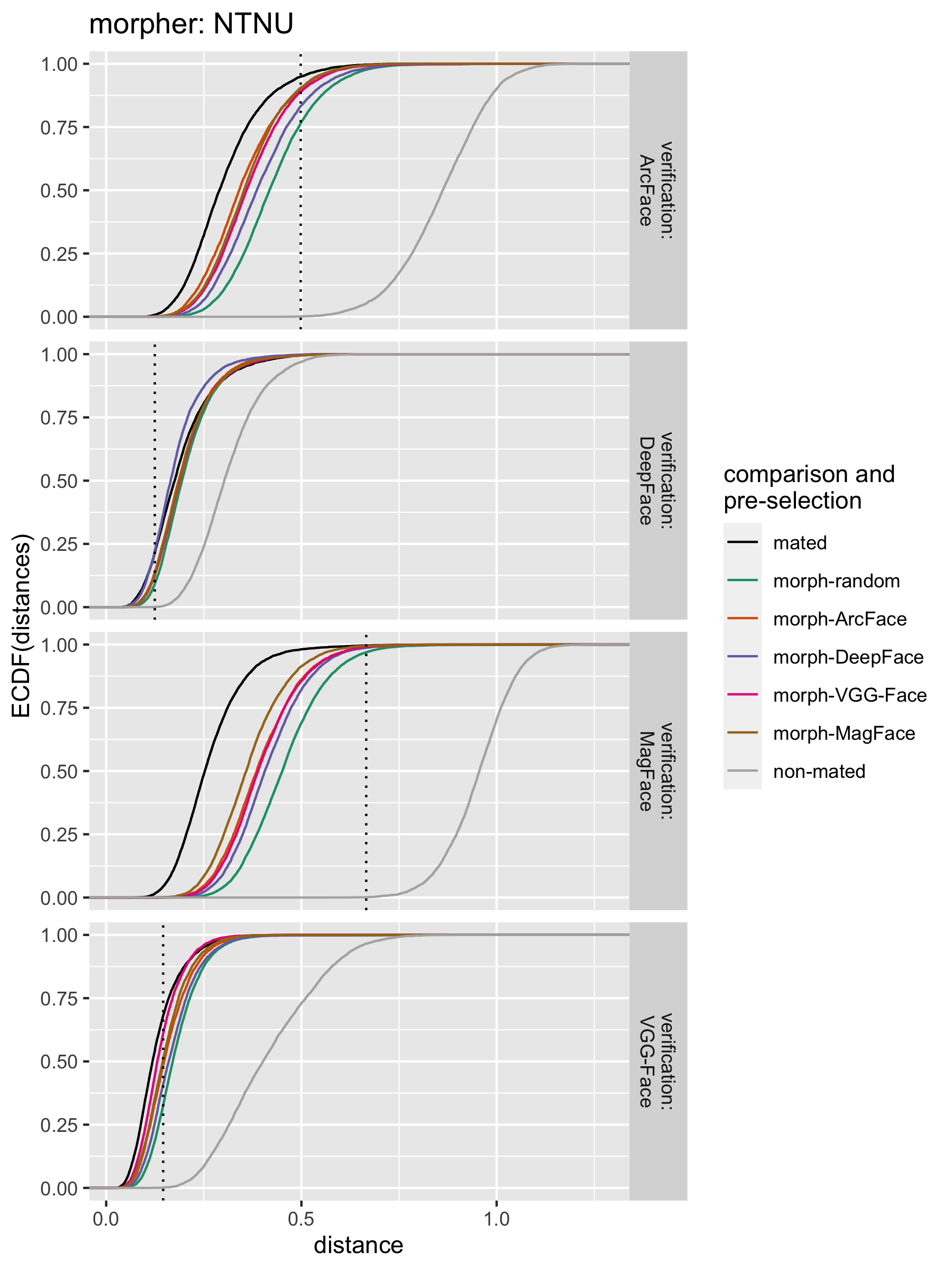

Supplement: S6 Fig — Morphs were created by NTNU morpher. See Fig 7 for details. (TIF) [file pone.0304610.s006.tif]

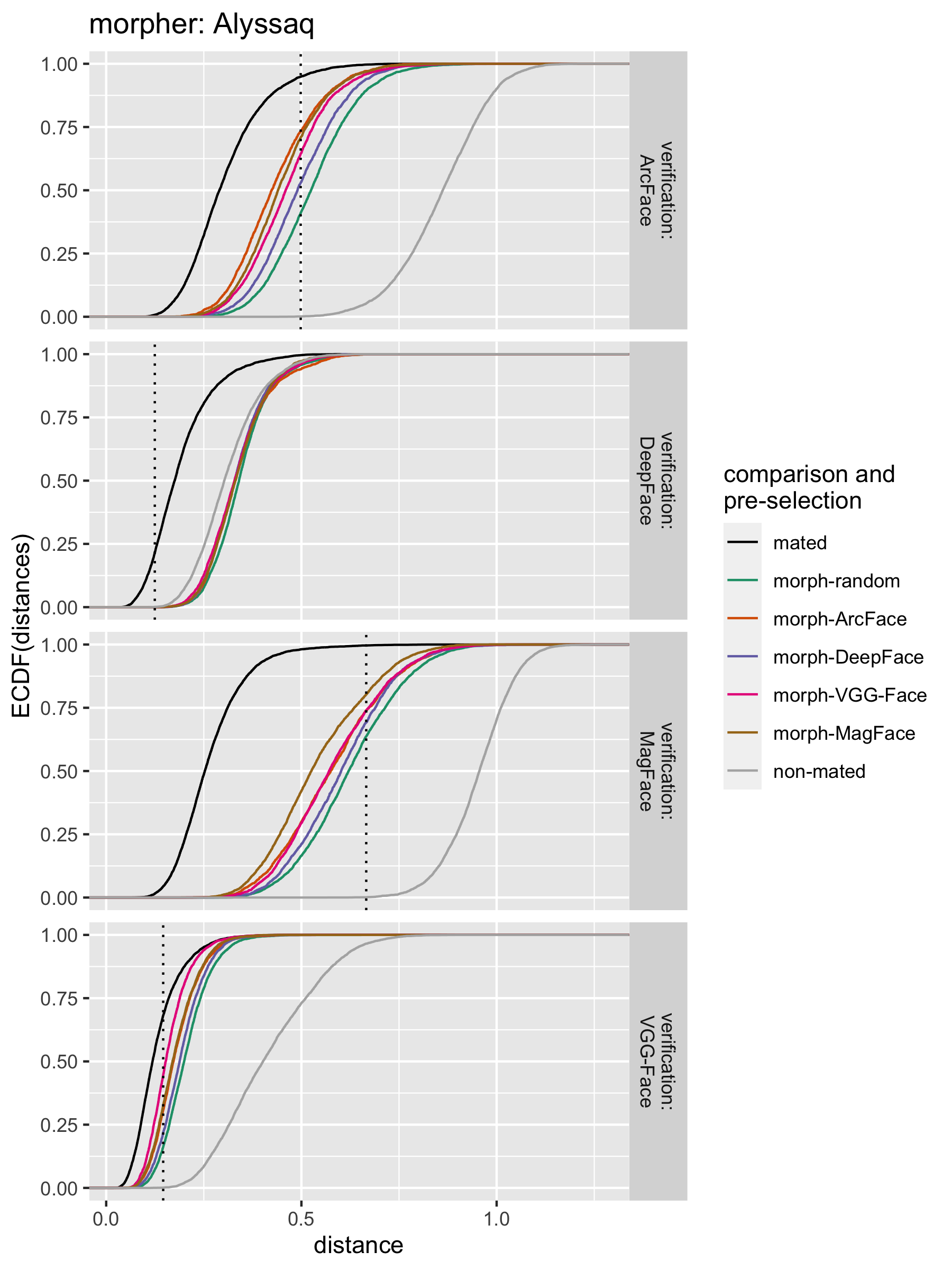

Supplement: S7 Fig — Morphs were created by Alyssaq morpher. See Fig 7 for details. (TIF) [file pone.0304610.s007.tif]

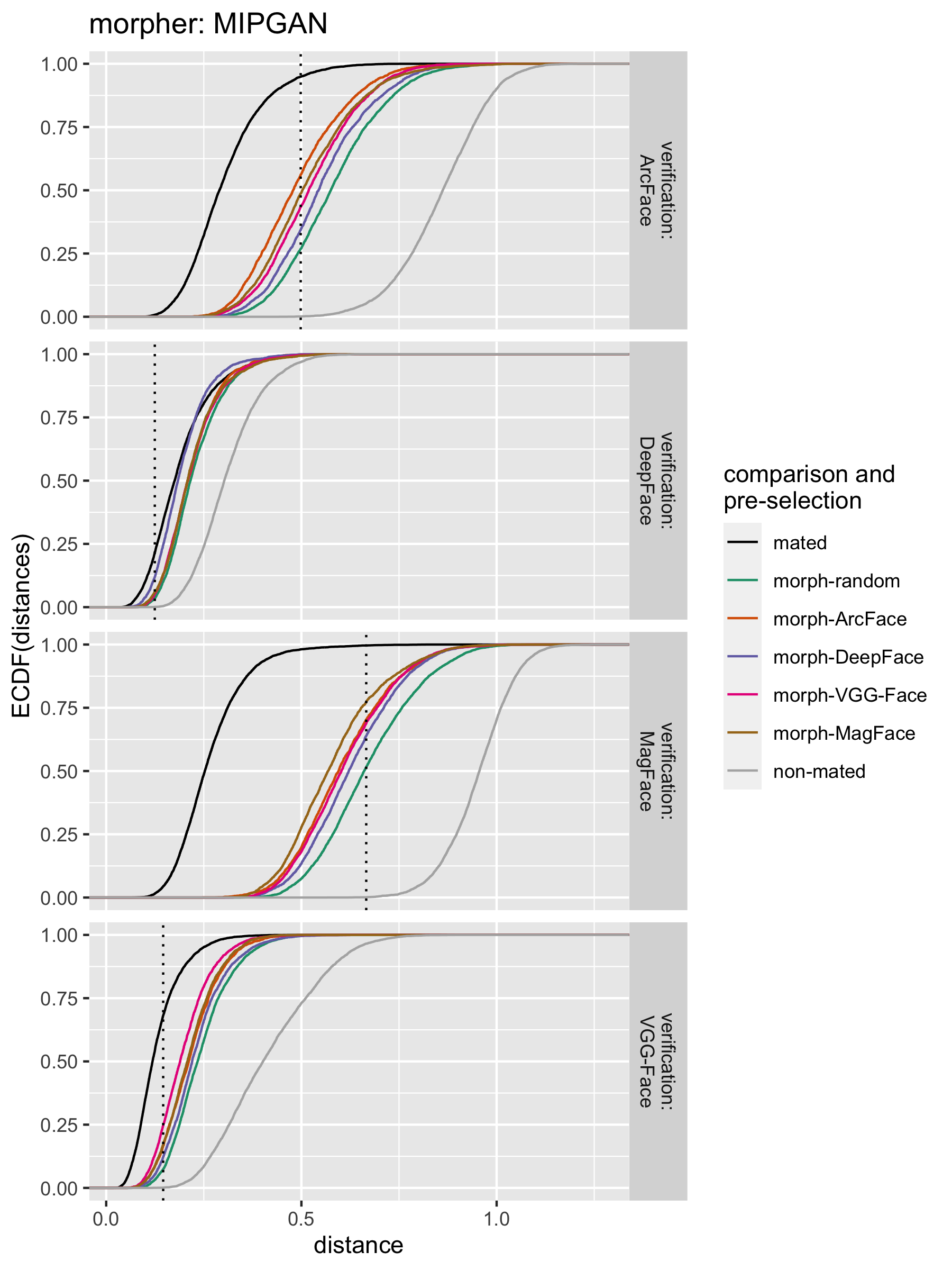

Supplement: S8 Fig — Morphs were created by MIPGAN morpher. See Fig 7 for details. (TIF) [file pone.0304610.s008.tif]

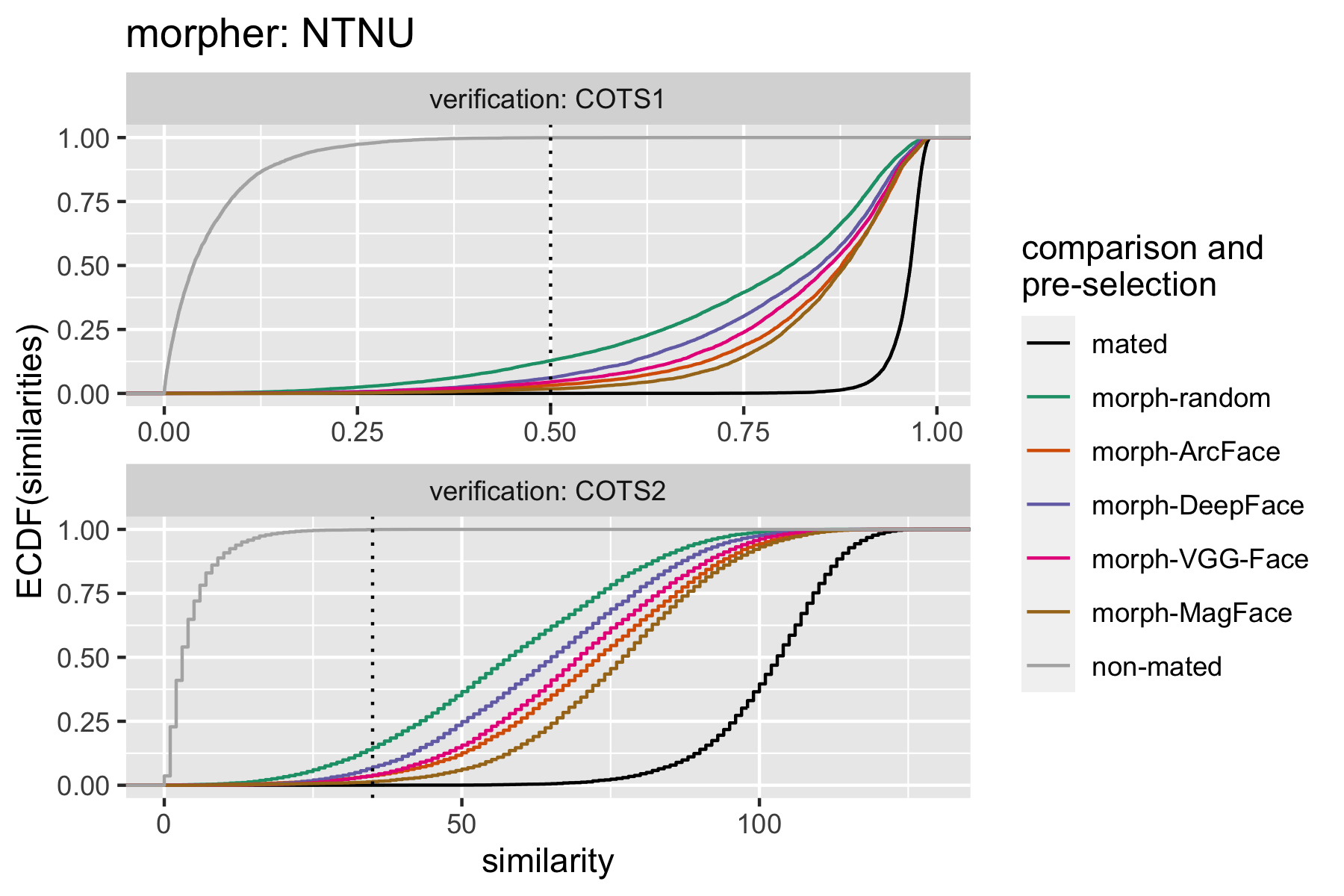

Supplement: S9 Fig — Morphs were created by NTNU morpher. See Fig 8 for details. (TIF) [file pone.0304610.s009.tif]

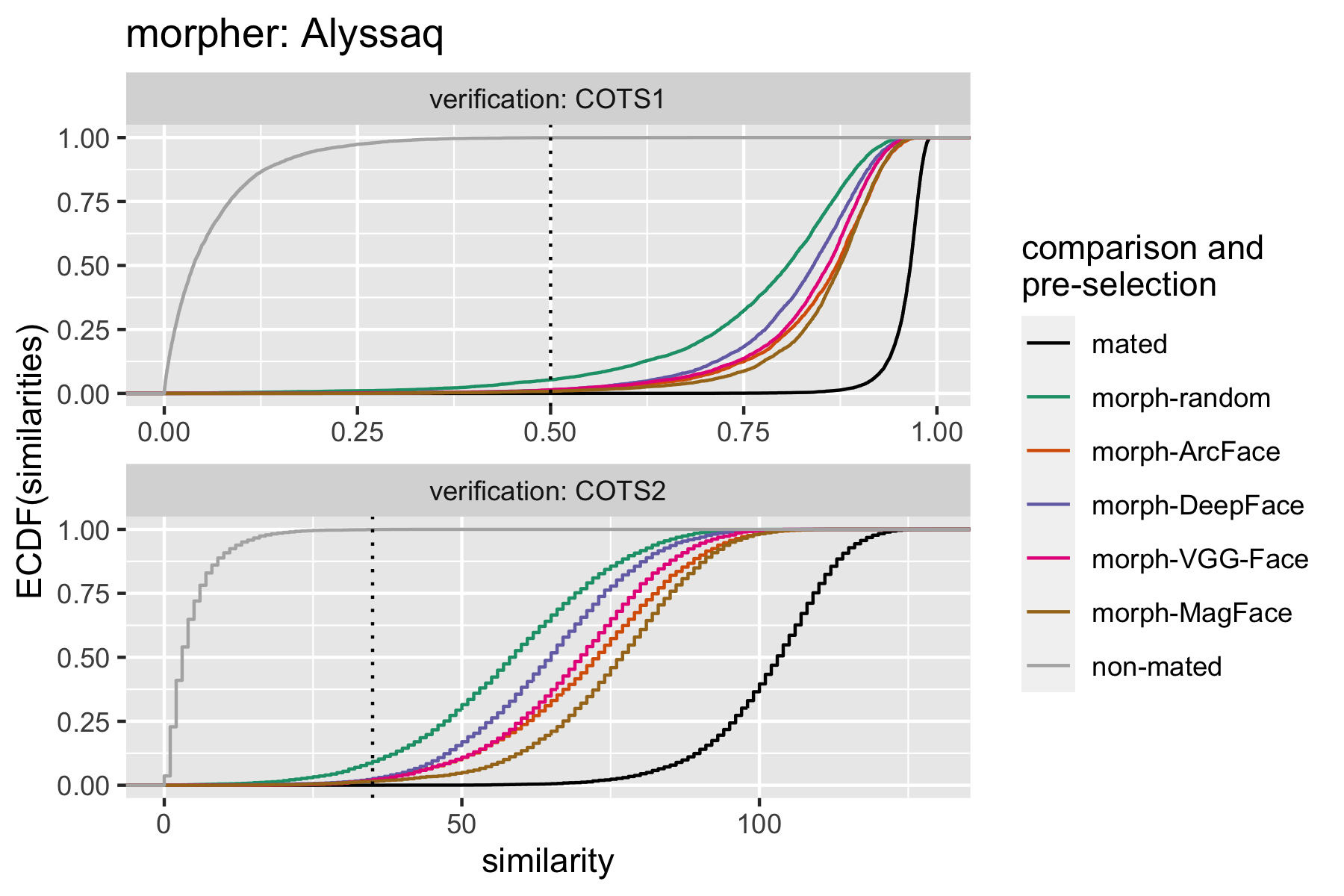

Supplement: S10 Fig — Morphs were created by Alyssaq morpher. See Fig 8 for details. (TIF) [file pone.0304610.s010.tif]

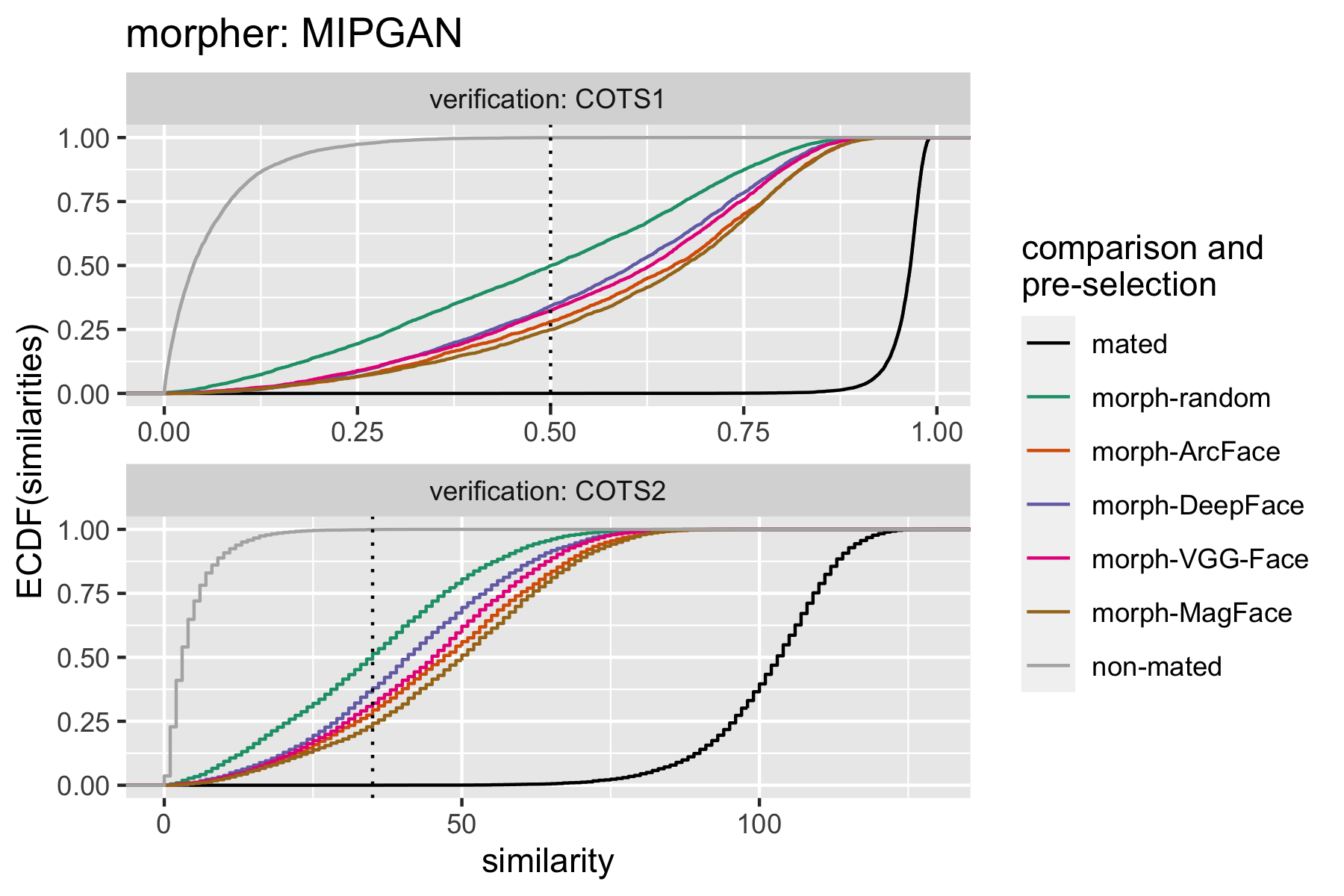

Supplement: S11 Fig — Morphs were created by MIPGAN morpher. See Fig 8 for details. (TIF) [file pone.0304610.s011.tif]

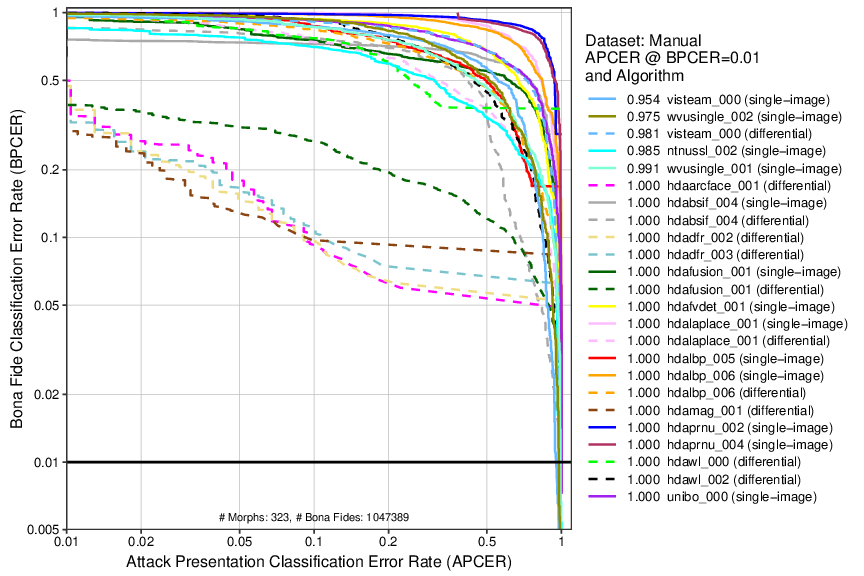

Supplement: S12 Fig — Figure used with permission. Inspired by the superior performance of MagFace over ArcFace for D-MAD in the present study, the proposed algorithm using MagFace embeddings has also been submitted to the Face Recognition Vendor Test (FRVT) MORPH of the National Institute of Standards and Technology (NIST) [45]. In this test, the detection accuracy of the submitted classifier using MagFace embeddings for D-MAD (named hdamag) was tested on morphed and bona fide face images of different data sets. Images of these data sets differed in various aspects, such as morph quality (automated vs. manually post-processed, publicly available tools, academic tools, or commercial tools) or with respect to the background, such as passport photographs vs. faces in natural environments. The figure illustrates the performance of the D-MAD algorithm on high-quality, manually post-processed morphed face images created with commercial morphing tools and high-quality, portrait-style bona fide images. The DET curves show good performance of the submitted hdamag algorithm. In particular, it outperformed all other algorithms in the evaluation with the lowest BPCER at the relevant security settings of APCER (i.e., MACER) values below 0.1. (TIF) [file pone.0304610.s012.tif]

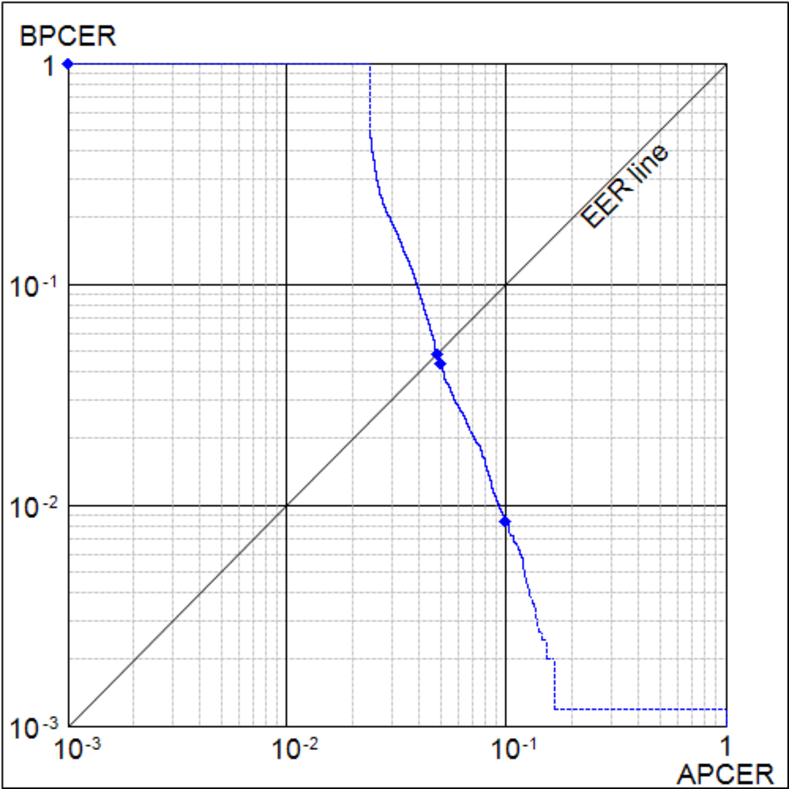

Supplement: S13 Fig — Figure used with permission. The MagFace D-MAD classifier has also been submitted to the FVC-onGoing section for Differential Morph Attack Detection [14, 46]. The detection accuracy of the submitted algorithm using MagFace embeddings for D-MAD (named hdamag) was tested on the DMAD-SOTAMD_P&S-1.0 benchmark. hdamag achieved lowest BPCER10 and second lowest BPCER20 values [47]. The DET curve is illustrated in log scale, with BPCER10, BPCER20, EER, and BPCER100 illustrated as blue dots. (TIF) [file pone.0304610.s013.tif]

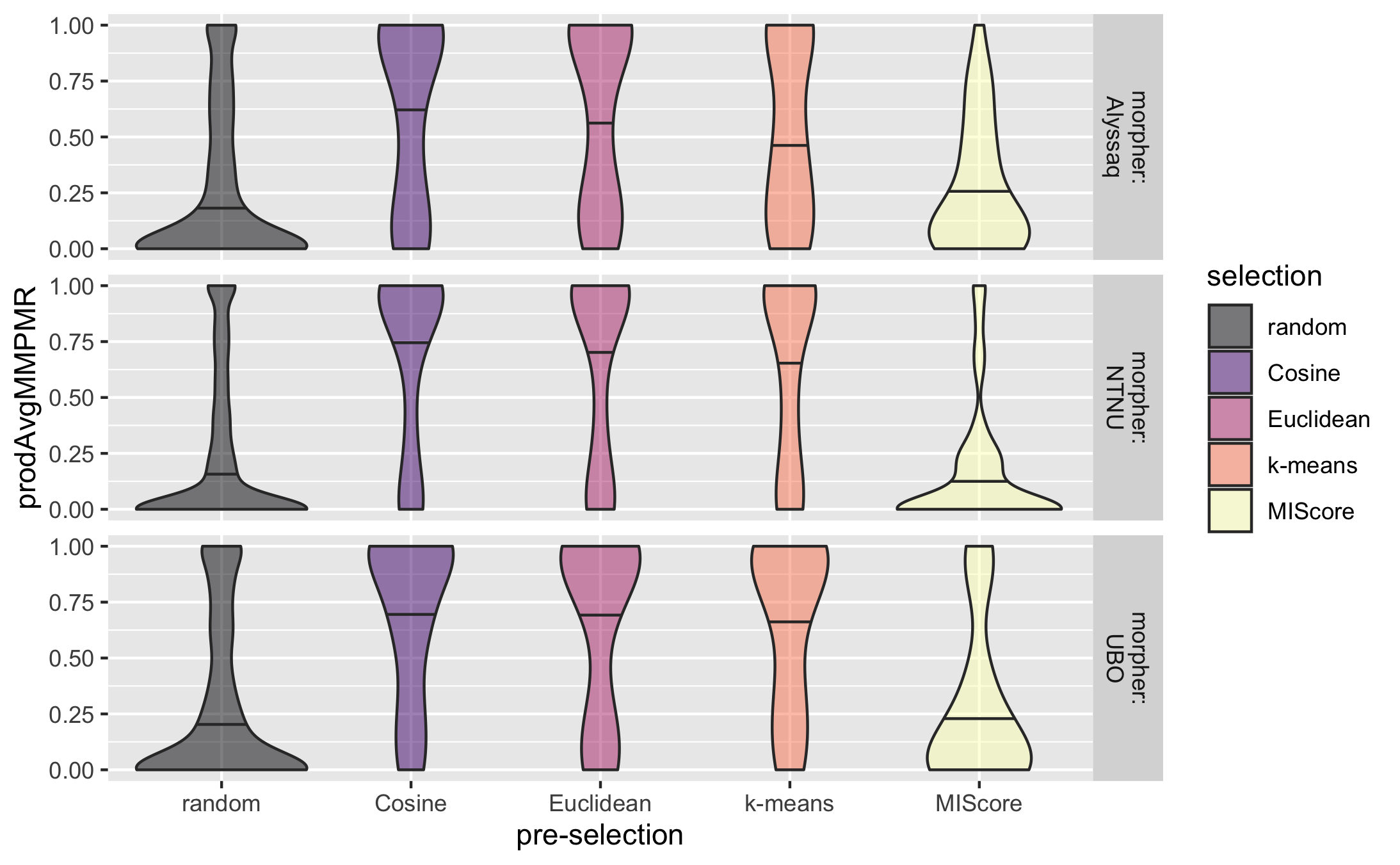

Supplement: S14 Fig — In a preliminary study, we investigated different distance measures and their influence on the attack potential of the resulting morphs. Images from the Face Recognition Grand Challenge database version 2 (FRGCv2) data set [49] were used for morphing. An in-house built subset of face images was used, comprising of 70 data subjects with around 10 captures each. We used different distance measures for pre-selection, such as Euclidean distance, Cosine distance, a distance measure based on the Mutual Information Score (miscore) [50], or alternatively a clustering algorithm that inherently uses Euclidean distance (k-means) [51]. Morphs based on pre-selection using Cosine distance achieved the highest performance in terms of attack potential, closely followed by the two measures based on euclidean distance. For this reason, we continued with Cosine distance metric for the pre-selection algorithm in the present study. (TIF) [file pone.0304610.s014.tif]
